# PAGER-CoV: a comprehensive collection of pathways, annotated gene-lists and gene signatures for coronavirus disease studies

**DOI:** 10.1093/nar/gkaa1094

**Published:** 2020-11-27

**Authors:** Zongliang Yue, Eric Zhang, Clark Xu, Sunny Khurana, Nishant Batra, Son Do Hai Dang, James J Cimino, Jake Y Chen

**Affiliations:** Informatics Institute, School of Medicine, The University of Alabama at Birmingham, Birmingham, AL 35223, USA; Informatics Institute, School of Medicine, The University of Alabama at Birmingham, Birmingham, AL 35223, USA; University of Wisconsin-Madison School of Medicine and Public Health, Institute of Clinical and Translational Research, Madison, WI 53705-2221, USA; Informatics Institute, School of Medicine, The University of Alabama at Birmingham, Birmingham, AL 35223, USA; Informatics Institute, School of Medicine, The University of Alabama at Birmingham, Birmingham, AL 35223, USA; Informatics Institute, School of Medicine, The University of Alabama at Birmingham, Birmingham, AL 35223, USA; Informatics Institute, School of Medicine, The University of Alabama at Birmingham, Birmingham, AL 35223, USA; Informatics Institute, School of Medicine, The University of Alabama at Birmingham, Birmingham, AL 35223, USA

## Abstract

**PAGER-CoV** (http://discovery.informatics.uab.edu/PAGER-CoV/) is a new web-based database that can help biomedical researchers interpret coronavirus-related functional genomic study results in the context of curated knowledge of host viral infection, inflammatory response, organ damage, and tissue repair. The new database consists of 11 835 **PAG**s (Pathways, Annotated gene-lists, or Gene signatures) from 33 public data sources. Through the web user interface, users can search by a query gene or a query term and retrieve significantly matched PAGs with all the curated information. Users can navigate from a PAG of interest to other related PAGs through either shared PAG-to-PAG co-membership relationships or PAG-to-PAG regulatory relationships, totaling 19 996 993. Users can also retrieve enriched PAGs from an input list of COVID-19 functional study result genes, customize the search data sources, and export all results for subsequent offline data analysis. In a case study, we performed a gene set enrichment analysis (GSEA) of a COVID-19 RNA-seq data set from the Gene Expression Omnibus database. Compared with the results using the standard PAGER database, PAGER-CoV allows for more sensitive matching of known immune-related gene signatures. We expect PAGER-CoV to be invaluable for biomedical researchers to find molecular biology mechanisms and tailored therapeutics to treat COVID-19 patients.

## INTRODUCTION

With COVID-19 becoming a pandemic, COVID-related biomedical research has generated a large amount of genomics and functional genomics data since January 2020 to characterize viral and host factors related to the disease outcome (1–5). As of 10 August 2020, the GEO database from the National Center for Biotechnological Informatics has reported 18 available COVID-19 genomic data sets in the GEO database (6) consisting of 73 samples using ‘COVID-19’ as the search term or 26 data sets consisting of 736 samples using ‘SARS-CoV-2’ as the search term (7). There is an urgent need to extract biological insights from SARS-CoV-2-related RNA-seq, single-cell RNA-seq and proteomic experimental results (2–5). Our ability to identify SARS-CoV-2 related genes, RNAs, proteins, interactions, functional network modules and pathways will help design new and better diagnostic techniques, therapeutic targets, or vaccines to fight against COVID-19 (7–9).

To perform functional genomics downstream analysis such as the Gene Set Enrichment Analysis (GSEA) (10), users today rely on general-purpose gene set databases, e.g. MSigDB (11), KEGG (12), EnrichR (13) or PAGER (14). However, while these databases generally contain ‘immune response’ pathways or gene signatures based on prior studies of cancer, autoimmune disorders, or other infectious diseases, they lack specific SARS-CoV-2 gene sets identified in recent SARS-CoV-2 genomic or functional genomic studies. For example, as of 1 August 2020, a quick search of ‘COVID’ or ‘SARS-CoV-2’ in MSigDB as of this publication returns no results and a search of ‘SARS’ or ‘coronavirus’ returns only one result. Likewise, a search using these queries against KEGG (12) retrieves only two COVID-19-related papers, while the same search against EnrichR returns no results. Increasing research has led to the development of several COVID-19 databases, e.g. the COVID-19 Drug and Gene Set Library (15) and the Databases for the targeted COVID-19 therapeutics (16), both of which were published in August 2020. However, these databases selected content covering only an incomplete aspect of the COVID-19 biomedical research topics and not all prior knowledge of immune response gene signatures and pathways from related immunological research studies. They also do not include computational analysis tools to help users perform gene set enrichment analysis. Therefore, to identify novel gene signatures and biological pathways as genomic features in various tissues due to viral infection remains an *ad hoc* exploratory process (17,18).

To provide the community with structured COVID-19 dedicated gene set data and a specialized GSEA search database, we developed **PAGER-CoV** (Pathways, Annotated gene-lists, and Gene signatures Electronic Repository for Corona Virus), accessible freely at http://discovery.informatics.uab.edu/PAGER-CoV/. For the current release of PAGER-CoV as of this publication, we compiled a total of 11 835 PAGs (Pathways, Annotated gene-lists, and Gene signatures) from 33 data sources including (i) expert-curated SARS-CoV-2 related PAGs from recently published high-quality COVID-19 papers in LitCoVID (19), (ii) curated COVID-19 pathways related to candidate drug repositioning candidates from the PubChem database (20) and (iii) selected immune response PAGs imported from the PAGER 2.0 database (14). PAGER-CoV is designed as a web database that compiles comprehensively curated gene sets on coronavirus-related infection, inflammation, organ damage, and repair from literature and public databases. PAGER-CoV has an intuitive user interface, with which users can perform both basic browsings of COVID-19 related **PAGs** using either a medical term such as ‘cytokine storm’ or an official gene symbol such as ‘ACE2’. Also, PAGER-CoV allows users to perform GSEAanalysis using a list of genes, e.g., those generated from a differentially-expressed gene list from a COVID-19 RNA-seq experiment, to quickly retrieve top-scoring PAGs that relate closely to the input gene lists. By browsing through retrieved PAGs, users can examine (i) virus or human gene components of each PAG, (ii) each PAG’s curated description, (iii) the source literature or database reference of each PAG, (iv) gene–gene interactions relationships among the genes covered by the PAG, (v) each PAG’s pre-calculated quality score (‘*nCoCo* Score’) that measures the PAG quality using topological intra-gene–gene interaction while controlling for PAG size (14) and (vi) related PAGs based on shared membership (m-type) or regulatory (r-type) PAG-to-PAG relationships described in (14,21). To accommodate the rapidly accumulating SARS-CoV-2 functional genomic data, we also designed a ‘Content Contribution’ page through which users can upload customized content for their incorporation into future releases. PAGER-CoV users can also download partial or full database content for advanced bioinformatics analysis elsewhere.

For the rest of this paper, we will describe how the database content was constructed, how web users could interact with the database, and why PAGER-CoV represents an improvement over the general-purpose gene set database for characterizing coronavirus-related functional genomics data.

## MATERIALS AND METHODS

### PAGER-CoV schema design and data source overview

**Figure 1.**
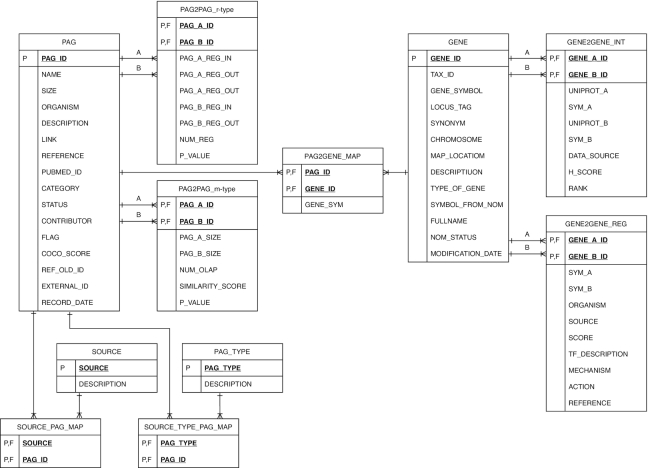
PAGER-CoV Schema. The *PAG* table represents the central element of the database; *SOURCE*, *SOURCE_TYPE* and *GENE* tables store additional information mapping to each PAG. There are 14 relationships among the 11 entities. The primary keys in the entities are underlined, bolded, and marked with ‘P’. The foreign keys in the entities are marked with ‘F’.

Figure 1 demonstrates the PAGER-CoV database schema, which contains eleven entities (also called tables) and fourteen relationships. The primary design was adapted from our prior work on the PAGER 2.0 database (14). Briefly, (i) the PAG table contains the general information of the PAGs, including the PAGs’ IDs, names, and data sources from which the PAGs are compiled, and PAG categories. As in (14). Each PAG belongs to either one of three categories: curated pathways/networks (P-type), curated gene sets without pathway/network (A-type), computationally derived gene sets with little or no curation (G-type), such as differentially expressed gene from an RNA-seq data. (ii) The GENE tables contains the general information of the genes, including names, official gene symbols defined by NCBI (https://ftp.ncbi.nih.gov/gene/DATA/GENE_INFO/), and external IDs linking to other well-known genetic databases. (iii) The *PAG-GENE MEMBER* table contains gene membership in each PAG. (iv) *GENE2GENE_INT* and *GENE2GENE_REG* tables contain the gene–gene interactions. Here, *GENE2GENE_INT* replicates the general protein–protein interactions in the HAPPI v.2.0 database (22); while *GENE2GENE_REG* replicates gene–gene regulations, which are validated in-vitro experiment, from the PAGER database (14). (v) The *PAG2PAG_R-TYPE* and *PAG2PAG_M-TYPE* tables contain two types of PAG-PAG relationships: regulatory and co-membership. As in (14) the PAG-PAG regulatory relationship reflects the PAG causal ordering inferred from gene-to-gene regulations; while the co-membership relationship reveals signaling cross-talk between PAGs that share signaling components within signal transduction pathways, in response to external stimuli. Data in the PAGER-CoV database is managed by the Oracle 19*c* relational database engine.

### Data collection overview

We compiled data into the PAGER-CoV database based on two general strategies: expert curation from literature and automated database integration. The expert curation involves manual data extraction from COVID-19 literature following by quality control, which is different from our earlier high-throughput automated software-based curation method (14,21).

### Curation of P-type PAGs from PubChem

To incorporate COVID-19 P-type PAGs, we performed web scraping for pathways relating to COVID-19 pathways on PubChem (https://pubchem.ncbi.nlm.nih.gov/#query=covid-19&tab=pathway). We wrote a Python 3 script on Anaconda distribution, which calls PubMed's Common Gateway Interface (CGI) (23) to download these PubChem COVID-19 pathways and their genes. The script directly made an API call to the PubMed website to get the most up-to-date gene expression of COVID-19 Pathways and refreshes on an automated batch schedule that maintains the data processing. Upon the downloaded pathway and gene information, the immunologist would curate, including revising the pathway description and removing COVID irrelevant genes, each pathway.

### Manual curation of A-Type PAGs

Four A-type PAGs representing computationally-predicted repositioned drugs for COVID-19 were curated from (24). Five A-Type PAGs were manually curated from Mouse Genome Informatics Database (MGI), reflecting tissue or cell development markers. For these PAGs from MGI, the mouse gene IDs were converted to official human gene symbols before being added to PAGER-CoV. An A-Type PAG representing cytokine-storm-related genes were curated from a review article (25). An A-Type PAG was generated by processing raw single-cell sequencing data from https://zenodo.org/record/3744141#.XuknTi2ZN24 and added to PAGER-CoV. Additionally, an A-Type PAG representing human exosome markers was curated from a review article (26).

### Literature curation of G-Type PAGs

Following comprehensive SARS-CoV-2 literature review, manual curation of SARS-CoV-2/COVID-19 G-Type PAGs from emerging SARS-CoV-2 literature or data source was performed using the following methodology. First, mapping of SARS-CoV-2 protein to SARS-CoV-2 gene information was manually curated from the NCBI GenBank database using the SARS-CoV-2 sequencing information (NCBI Reference Sequence: NC_045512.2) isolated from patient zero at the Wuhan Seafood Market in Wuhan, CN (27). SARS-CoV-2 gene symbols were mapped to the viral protein product, e.g. ‘ORF1ab polyprotein’ mapped to the ORF1ab gene. G-Type PAGs manually curated from this study were given appropriate PAG Titles (e.g. ‘Viral gene encoding SARS-CoV-2 Nsp1 viral protein’ for SARS-CoV-2 protein nsp1), and annotated with additional information in the ‘PAG Name’ field. Mature peptide sequence information was matched to corresponding viral gene or open reading frame product information, alongside corresponding protein IDs. Annotation of the SARS-CoV-2 protein function, e.g. ‘Geneset description’ attribute, was taken from the COVID-19 subset of the UniProtKB database (28). A total of 33 PAGs (each containing a single viral gene member) were compiled in this manner, representing the relationship between viral proteins and the viral gene.

Following this step, PAGs relating to in-vitro-validated SARS-CoV-2 viral protein to human host gene interactions were curated from a study where the authors cloned and expressed SARS-CoV-2 viral proteins in-vitro and identified human host binding partners using affinity purification mass spectrometry (29). A total of 88 PAGs were curated from this study—71 PAGs representing the total viral-to-human protein-protein binding partners identified, and 17 PAGs representing known druggable targets. In addition, 64 PAGs representing the significant cellular pathways disrupted during SARS-CoV-2 infection were curated from another proteomics study in which authors used human cell-culture lines to examine proteomic changes in SARS-CoV-2 infected human cell-lines over time (2).

Next, we curated repositioned drug target gene-sets relating to clinical drugs under investigation to treat COVID-19. COVID-19 repositioned drugs, and their associated human protein drug targets and ADME proteins, were manually curated from the DrugBank database (30). Missing genes from the DrugBank database were manually searched for in literature and cited accordingly. PAGs with missing genes were excluded from import into PAGER-CoV. From this step, a total of 96 completed drug target/ADME-associated G-Type PAGs were added to PAGER-CoV.

For the final step of manual curation, available raw sequencing data from newly emerging COVID-19 studies was searched on the NCBI GEO database with keyword search terms ‘COVID-19’ and ‘SARS-CoV-2’. Available datasets were comprehensively evaluated by our curation team to identify high-quality COVID-19-specific G-type PAGs and were processed, analyzed, and curated into PAGER-CoV by our curation team. To compare host-related immune responses in patients between SARS-CoV-2 and other respiratory viruses, raw RNA-sequencing data available from clinical samples of non-SARS-CoV-2-related viral pneumonia were also re-analyzed, processed, and added to PAGER-CoV as two separate PAGs (31). Therefore, a total of ten G-type PAGs were collected this way.

### Integrating indirectly related PAGs from PAGER

Since SARS-CoV-2 is a new coronavirus that shares many biological mechanisms of infection and immune response profiles in tissues with other viral infections, in this step, we seek to integrate ‘indirectly-related’ PAGs from the existing gene set database into PAGER-CoV. We decided upon using our previously published PAGER 2.0 database, because (i) PAGER 2.0 incorporates a wide array of heterogeneous data sources for comprehensiveness (e.g. MSigDb, BioCarta, DSigDb, as well as deprecated data sources such as GeneSigDb), (ii) PAGER 2.0 contains thoroughly-curated gene sets from validated, high-quality data sources with additional annotations and (iii) PAGER 2.0 is structured ease of comparison due to construction of quality measures for PAGs (i.e. *nCoCo* score). To import relevant PAGs from PAGER 2.0 to PAGER-CoV, we used the following search terms related to host viral infection, inflammatory response, organ damage, and tissue repair: ‘viral’, ‘virus’, ‘infection’, ‘inflammation’, ‘immunity’, ‘tissue repair’, ‘organ repair’, ‘inflammatory’, ‘Tcell’, ‘Bcell’, ‘T-cell’, ‘B-cell’, ‘monocyte’, ‘interferon’, ‘CD4’, ‘CD8’, ‘Treg’, ‘immune response’, ‘toll like receptor’, ‘TLR’, ‘oxidative stress’, ‘interleukin’, ‘tissue damage’, ‘regeneration’, ‘vitamin D’, ‘chemokine’, ‘hypoxia’, ‘TNFalpha’, ‘NF-kappaB’, ‘LPS’, ‘cytokine’, ‘peripheral blood mononuclear cells’, ‘pbmc’, ‘leukocyte’, ‘granulocyte’, ‘neutrophil’, ‘monocyte’, ‘lymphoid’, ‘lymphocyte’, ‘vaccine’, ‘vaccination’, ‘dendritic’, ‘inflammatory’, ‘wound healing’, ‘CD34’, ‘interferon’, ‘interleukin’, and ‘macrophage’. In total, 10 015 PAGs covering 18 907 genes were imported from PAGER 2.0.

### PAG data quality control

To clean the data from the curated source, we created an automatic checking system to correct errors in curated data, assigning the internal PAG identifiers and insert into the PAGER-CoV database. We observed that the errors came from three aspects, the first type of failure coming from curation, such as duplicate genes in a PAG member list or invalid genes with no official gene names or Entrez IDs that needed to be fixed. The second type of error is invalid characters embedded in contents, such as u’\xa0’ was replaced by space, u’\u2030′ was replaced by ‘&quote’ etc. The third type of error is the missing annotations in original data, such as a few pathways in PubChem, which had no taxonomy name. We pulled out these pathways, manually checked pathway description and information in original sources, add added back the species. To assign new identifiers to PAGs in sequence, we characterized the type of the PAGs using three-letter in the naming convention, retrieved the last number of existing type-specific PAGs in the database, and assembled the new identifier. Before inserting the records, our curator team validated and approved each PAG individually

### Additional PAG annotations

The quality of PAGs is measured by a normalized statistically significant coverage of gene-gene functional correlations in gene-pairs or gene-triplets, named ‘normalized Cohesion Coefficient score (*nCoCo*)’ in PAGER 2.0 (14). The quality of PAGs is measured by a normalized statistically significant coverage of gene-to-gene functional correlations in gene-pairs or gene-triplets, named ‘normalized Cohesion Coefficient score (*nCoCo*)’ in PAGER 2.0. The brute force way of measuring the quality of PAGs is to report a total count of all the interactions for each PAG. However, it does not provide measurements against the background, and such count can vary dramatically when other non-quality factors change, e.g. increase of PAG size. Therefore, we introduce *nCoCo* score to address the following problems:

1. In *nCoCo* score, we measure not only the count of ‘binary interactions’ but also ‘interaction triangles’, the latter of which is a measure of the existence of network modules.
2. In *nCoCo* score, we convert the count of interactions and interaction triangles into a statistic against the count in the background distribution from randomly generated PAGs. Therefore, the reported statistic carries more statistical significance than a simple count.
3. In *nCoCo* score, we perform additional size normalizations (method described in PAGER 2.0) to make the density score of PAGs at varying sizes comparable by eliminating the score's size bias.

The gene prioritization within PAGs is based on gene weight calculated in the PAG, called 'relevant protein score (RP-score)' was described in PAGER 2.0 (14).

To compute the *nCoCo* scores, first, we applied the HAPPI-2 database to recalculate the *CoI* and *CoT* scores using the hypergeometric cumulative distribution function (CDF). Second, we build the multi-box plots using the bins with log_2_-scale of PAG gene sizes and used the median to represent the value in each bin and applied the polynomial function to find the regression of the *CoI* score vs PAG size.

(1) $$\begin{equation*}CoI\left( p \right) = Sz{\left( p \right)^2}*a + Sz\left( p \right)*b\end{equation*}$$

where *Sz(p)* is the size of the PAG *p*, and the *CoI(p)* is the *CoI* score of the PAG *P*.

Third, we calculated *nCoCo* score based on the formula:

(2) $$\begin{eqnarray*} && { nCoI}\left({ p} \right) = { med}\left({{ PA}{{{ G}_n}}} \right) \nonumber \\&& *{ CoI}\left({ p} \right) /\left[ {{ Sz}\left({ p} \right)\ *{ a} + { Sz}{{\left({ p} \right)}^2}*{ b}} \right] \end{eqnarray*}$$

where $med( {PA{G_n}} )$ is the median gene size of all PAGs. *a* and *b* are coefficients.

Fourth, the *nCoCo* score is calculated by the sum of the normalized interactive score *nCoI* and normalized triangle score *nCoT*:

(3) $$\begin{equation*}nCoCo\left( p \right) = nCoI\left( p \right) + nCoT\left( p \right)\end{equation*}$$

To find an optimal *nCoCo* score cutoff, we created a negative set of PAGs by substituting gene members in ‘true’ PAGs with gene members randomly generated from the PAGER-CoV database. After calculating the *nCoCo* score of the negative PAGs, we chose the optimal *nCoCo* score cutoff that maximized the product of sensitivity (true positives over true cases) and specificity (the true negatives over negative cases).

### PAGER-CoV database web user interface

The web user interface implemented the following essential functionalities for biomedical researchers and bioinformaticians: (i) **Basic Search**. On the main home page, users can search the database using a medical term or a gene symbol and retrieve a list of PAGs. The retrieved PAGs can be refined, explored on the web, or downloaded onto the user's computer for further analysis. (ii) **Downstream analysis**. On the ‘Analyze’ page, users can perform GSEA with an input gene list. Users can customize the statistical parameters according to the user's specific experimental requirements. (iii) **Contribute content**. On the ‘Contribute’ page, a user can upload their curated gene sets and pathways for review and subsequent consideration for inclusion into the PAGER-CoV database. The submission file could be either differential gene expression format (DEG) or literature-curation format (LIT), as described on the ‘Contribute’ page. After submission, the contributed data will be checked for quality and eventually integrated into the PAGER-CoV after passing quality checks. (iv) **Download the database**. On the ‘Download’ page, users can download different database versions. This feature allows users to perform independent GSEA analysis. PAGER-CoV is free and open to all users, and there is no login requirement.

The PAGER-CoV website features an improved user interface and user-upload schema over the related PAGER 2.0 database, with a more intuitive user-side browsing, analysis, and submission experience (Figure 3). To improve user navigations, we restructured the PAGER web interface to have the ‘Basic Search’ function as the feature-in-focus on the PAGER-CoV home page. We also streamlined the navigation from one PAG to related PAGs, by adding a ‘related PAGs’ box to the right of each PAG’s summary content.

### Data processing related to the case study

To show that PAGER-CoV improves COVID-19 functional genomics analysis, we compared the GSEA (10) results between two conditions: one using PAGER 2.0 as the reference pathway/gene set collection, the other using PAGER-CoV as the reference pathway/gene set collection. We selected the ‘Transcriptional response to SARS-CoV-2 infection’ from GEO data series (ID: **GSE147507**) (32) for the case study. In the step of data filtering, all four control samples from the ‘NHBE_Mock’ and three ‘NHBE_CoV’ experimental samples were processed in parallel using the DESeq2 (33) pipeline. Then, we performed standard GSEA analysis (10) by comparing the results using the PAGER-CoV database (release date: 3 August 2020) and the results using the standard PAGER 2.0 database (14). For the GSEA analysis, the GSE147507 downloadable files for normalized gene expression matrix and the sample label file ‘GSE147507.all.label.gsea.cls’ were used (Supplemental File S1). GSEA chip platform choice ‘ftp.broadinstitute.org://pub/gsea/annotations_versioned/Human_Symbol_with_Remapping_MSigDB.v7.1.chip’ were used, whereas all other parameters were set to GSEA software (https://www.gsea-msigdb.org/gsea/downloads.jsp) default. For candidate PAGs for GSEA analysis, we used only PAGs with gene sizes between 15 and 500. After filtering, 18 136 candidate PAGs in PAGER 2.0 and 4 612 candidate PAGs in PAGER-CoV remained.

## RESULTS

### PAGER-CoV data compilation and data quality assessment

In PAGER-CoV, we compiled a total of 11 835 PAGs from 33 data sources. Table 1 shows a summary of PAG counts categorized by the data source. There are 13 data sources covering 271 PAGs manually curated from SARS-CoV-2 literature or relevant databases, 1 549 PAGs web-scraped from the COVID-19 PubChem database, and 19 PAGER 2.0-inherited data sources comprising 10 015 viral and immune-related PAGs inherited from PAGER 2.0.

**Table 1.** PAGER-CoV PAG count and Data Sources. PAGER-CoV consists of three major source categories: (i) curated PAGs inherited from the original PAGER database (PAGER); (ii) PAGs curated from the PubChem COVID-19 Pathway database (PubChem); (iii) PAGs manually-curated from selected SARS-CoV-2-related literature or database resource (curation)

Category	Source	Count	
	PAGER-BioCarta	105	
	PAGER-DSigDB	49	
	PAGER-GAD	70	
	PAGER-GOA	1888	
	PAGER-GOA_EXCL	1030	
	PAGER-GTEx	2	
	PAGER-GWAS Catalog	79	
	PAGER-GeneSigDB	390	
	PAGER-KEGG	38	
*PAGER (ver. 2.0)*	PAGER-MSigDB	6139	10015
	PAGER-NCI-Nature Curated	13	
	PAGER-NGS Catalog	1	
	PAGER-Pfam	82	
	PAGER-PharmGKB	4	
	PAGER-PheWAS	57	
	PAGER-Protein Lounge	30	
	PAGER-Reactome	25	
	PAGER-Spike	3	
	PAGER-WikiPathway	10	
*PubChem*	PubChem pathway	1549	1549
	Am J Respir Crit Care Med	2	
	Cell	5	
	Cell Host and Microbe	1	
	Drugbank	96	
	GenBank (gene mapping), COVID-19 UniProt (for Geneset Description)	33	
	Microbiology and Molecular Biology Reviews	1	
*Curation*	Mouse Genome Informatics Database	5	271
	Nature	111	
	Nature Cell Discovery	4	
	Nature Medicine	1	
	The Annual Review of Cell and Developmental Biology	1	
	Zenodo	1	
	bioRxiv	10	
Total		11835	

**Figure 2.**
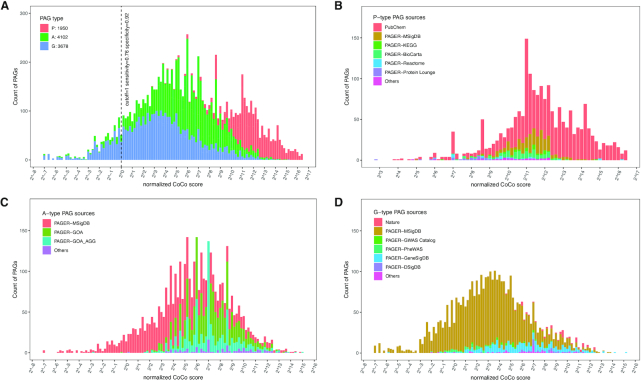
PAGER-CoV Data Quality PAGER-CoV data quality distribution; *nCoCo* score distribution breakdown by PAG-type. (**A**) *nCoCo* score distribution of the three PAG types. Dashed line represents the optimal *nCoCo* score cutoff (= 1) with sensitivity = 0.76 and specificity = 0.92. (**B**) P-type PAG *nCoCo* score distribution grouped by sources. The ‘Others’ category includes ‘PAGER-NCI-Nature Curated’, ‘PAGER-WikiPathway’, ‘PAGER-PharmGKB’ and ‘PAGER-Spike’. (**C**) A-type PAG *nCoCo* score distribution grouped by sources. The ‘Others’ category includes ‘Nature’, ‘Nature Cell Discovery’, ‘The Annual Review of Cell and Developmental Biology’, ‘Microbiology and Molecular Biology Reviews’, ‘Drugbank’, ‘Zenodo’, ’Mouse Genome Informatics Database’, ‘bioRxiv’, ‘PAGER-Pfam’ and ‘PAGER-GTEx’. (**D**) G-type PAG *nCoCo* score distribution grouped by source. The ‘Others’ category includes ‘Am J Respir Crit Care Med’, ‘PAGER-GAD’, ‘Nature Medicine’, ‘Cell’, ‘Cell Host and Microbe’ and ‘PAGER-NGS Catalog’.

Figure 2 shows the *nCoCo* score distribution for all the PAGs (P-type, A-type, and G-type) distributed over different score intervals. Since *nCoCo* score is a measure of PAG data curation quality (see the Materials and Methods section for details), we can compare the relative distribution of PAGs over *nCoCo* score intervals to determine how biologically ‘informative’ these PAGs can be. The quality score distribution result indicates that P-type PAGs in PAGER-CoV has the highest quality (*nCoCo* score mean = 8 126), followed by A-type PAGs as the second-highest (*nCoCo* score mean = 338), and followed by G-type PAGs as the lowest (*nCoCo* score mean = 155). However, the majority (92%) of all PAGs has a quality no less than the quality score cutoff ( = 1).

### PAGER-CoV web-based search interface

**Figure 3.**
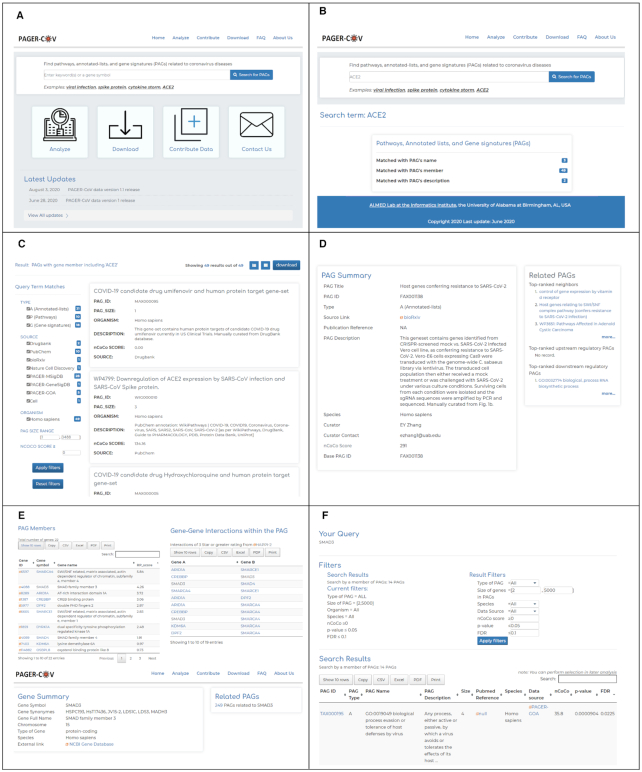
PAGER-CoV Web Interface and Basic Search Case Study. (**A**) Homepage of PAGER-CoV webserver. (**B**) The summary page of retrieved PAGs using the keyword ‘ACE2’ (**C**) The page of retrieved PAG results after clicking on the ‘matched with PAG’s member – 49’. The left panel is ‘query term matches,’ which allows users to filter the PAGs based on the PAG attributes. The right panel is the itemized overview of retrieved PAGs. (**D**) The PAG detail page after tapping a PAG name. The PAG summary on the left side contains the PAG detailed information with outsourcing links. The related PAGs on the right side provides the top-ranked PAGs evaluated by m-type and r-type relationship scores. The full ranked PAG list can be retrieved by clicking on the ‘more…’ (**E**) The GENE detail page after clicking on a ‘gene symbol’ (e.g. SMAD3). The gene summary composites the gene detailed information and an NCBI link. (**F**) The page of ‘Related PAGs’ retrieved result. There are multiple PAG attributes allowing users to filter out uninteresting PAGs.

Figure 3A-F demonstrate a typical searching session in PAGER-CoV. In Figure 3A (basic search), the user may enter a search term, such as ‘spike protein’, ‘cytokine storm’, ‘ACE2’, or ‘TMPRSS’. Figure 3B shows the basic search result. Here, the ‘ACE2’ result contains 53 PAGs; 49 PAGs contain ACE2 genes (matched by ‘member’), and 2 PAGs have ‘ACE2’ in the PAG description (matched by PAG description). Figure 3C shows the list of PAGs, sorted by the PAG size, when ‘match by member’ is selected. Selecting ‘batched by PAG description’ shows a similar result. Here, the user may also filter the PAG list by PAG Type, Source, and Organism. Figure 3D shows the PAG information when a specific PAG is selected. From here, the user can view which genes the PAG contains (Figure 3E), how important each gene is in the PAG (quantified and sorted by the RP-score), and the relationship with other PAGs (Figure 3F). By using PAGER-CoV as a comprehensive database for interactive browsing, researchers can quickly gather gene set information, identify related literature, and generate new hypotheses.

### PAGER-CoV reveals insights of how bronchoalveolar immune cells response to COVID-19

**Figure 4.**
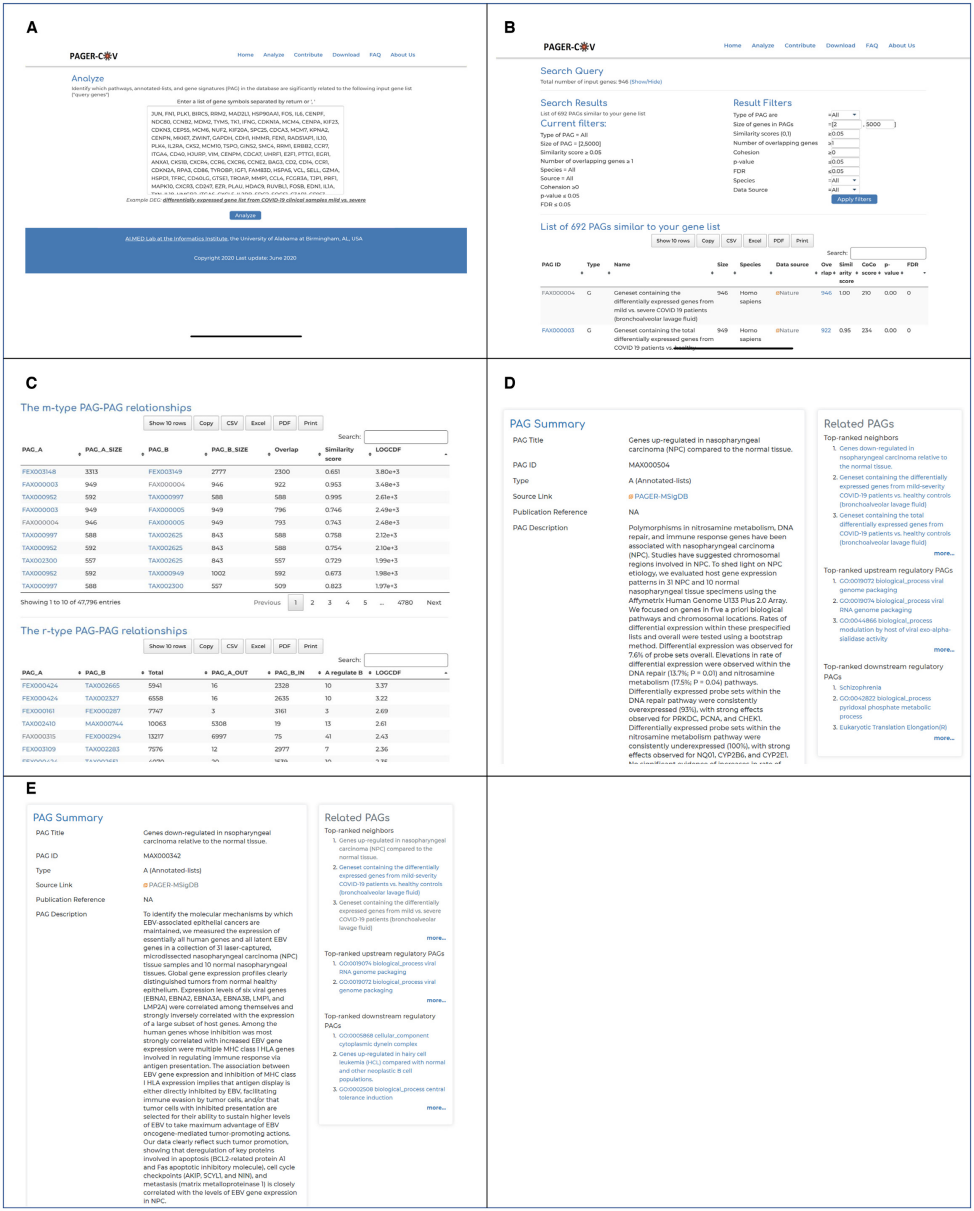
PAGER-CoV Analysis Case Study and User-Submitted PAGs. (**A**) An example of the input gene list ‘differentially expressed gene list from COVID-19 clinical samples mild vs. severe’. (**B**) The page of retrieved PAG results. (**C**) M-type and r-type PAG-PAG relationship information below the ‘Retrieved PAG results’ page. (**D**) PAG detail page after tapping on a PAG ID. (**E**) Example of retrieved top-ranked neighboring PAGs ‘Genes down-regulated in nasopharyngeal carcinoma relative to the normal tissue.’ of the PAG ‘Genes up-regulated in nasopharyngeal carcinoma (NPC) compared to the normal tissue’.

Since the lung is among the most common organ attacked by COVID-19, there have been many studies investigating the lung response to COVID-19. Therefore, we are interested in analyzing the single-cell transcriptomic data under COVID-19 using PAGER-CoV. Here, we processed raw single-cell RNA-seq data from the GEO database GSE145926 data set. The data set were collected from clinical bronchoalveolar lavage fluid samples from moderate vs. severe cases of COVID-19 (34). The significant differentially-expressed gene list that was computed using the Seurat pipeline (35) was used in the PAGER-CoV GSEA analysis. PAGER-CoV provided 692 PAGs (Figure 4A–C) with the default cut-offs as follows: ‘type of PAG‘ is set to ‘all’, ‘size of genes in PAGs’ ranges from 2 to 5 000, ‘similarity score’ ≥ 0.05, ‘number of overlapping genes’ ≥ 1, ‘nCoCo’ ≥ 0, ‘*P*-value’ ≤ 0.05, ‘False Discovery Rate’-adjusted *P*-value (FDR) ≤ 0.05, ‘species’ is set to ‘all’, and all ‘data sources’ are selected. Among the top ten results retrieved by FDR, all are directly related to coronavirus infections, eight of which are manual curated PAGs. Interestingly, two (MAX000504, MAX000342) of the ten top-ranked PAGs were imported from PAGER from the same study (36), which are up-regulated and down-regulated gene sets in response to Epstein-Barr Virus (EBV) infection in individuals with nasopharyngeal carcinoma epithelial cancer (Figure 4D). Other neighboring PAGs related to MAX000504 may also have major roles in the COVID-19 immune response. For example, GEX000051, a top-ranked downstream regulatory PAG for MAX000504, was shown as derived from a ‘genome-wide association study of maternal cytomegalovirus infection and schizophrenia’ (37). This molecular gene set evidence confirms the potential linkage between COVID-19 and the psychiatric and neurological effects of SARS-CoV-2 infected patients, which reported the clinical observation of COVID-19 Psychosis in many patients (38) (39). Meanwhile, although MAX000342 is indirectly related to this study, the 277 down-regulated genes identified from Epstein-Barr Virus (EBV)-associated nasopharyngeal carcinoma epithelial cancer tissue samples contain the host MHC Class I HLA gene family members (40). Susceptibility to COVID-19 severity based on immune MHC haplotype is an area being actively investigated (41) and supported by increasing evidence (42). Other downstream regulatory PAGs to MAX000342 are reported by PAGER-CoV (Figure 4E). Users can download the search results and explore PAGs further with their own desktop computers.

### PAGER-CoV enhances GSEA analysis in COVID-19 specific study

**Figure 5.**
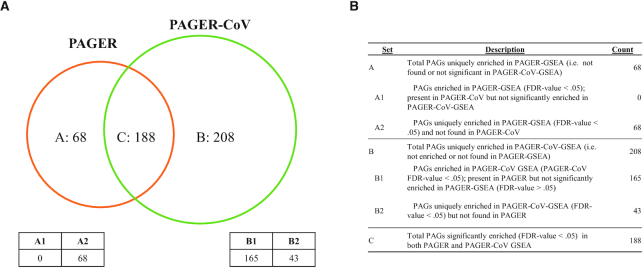
PAGER-CoV versus PAGER-original Comparison. (**A**) Venn diagram of PAGER-CoV GSEA versus PAGER-GSEA analysis results. (**B**) Tabular breakdown of Sets A and B. Further detailed annotations of Set A, Set B, Set C, Set A1, Set A2, Set B1 and Set B2 can be viewed in Supplementary Table S2.

Using the differentially express genes in GSE147507 dataset as the input, our results show that GSEA supported by PAGER-CoV is better than the same analysis supported by general-purpose gene set databases such as PAGER 2.0 (Figure 5, Supplemental File S2). Between 396 enriched PAGs from the PAGER-CoV-GSEA results and 256 enriched PAGs from the PAGER-GSEA results, there are 188 ‘**Set C**’ shared PAGs (FDR *q*-value ≤ .05). In PAGER-CoV-GSEA, there are 208 unique PAGs (‘**Set B**’), consisting of 165 PAGs derived from the PAGER-imported subset (‘**Set B1’**) and 43 PAGs derived from a newly curated subset only in PAGER-CoV (‘**Set B2’**). We manually examined the 165 Set B1 PAGs and found all of them to be of high biological relevance to SARS-CoV-2, including 7 already confirmed by additional SARS-CoV-2 literature. In PAGER-GSEA, on the other hand, contains only 68 PAGs uniquely identified in the PAGER 2.0 database (‘**Set A2**’) and 0 PAGs derived from imported PAGER-CoV (‘**Set A1**’). We manually examined the 68 Set A2 PAGs and found only 9 to be of high biological relevance to SARS-CoV-2, 45 to be of possible biological relevance, and 14 to be of little direct biological significance. This comparison results show that using PAGER-CoV for GSEA can not only pick up newly curated PAGs but also help improve the sensitivity of detection for existing imported PAGs, i.e., B1 PAGs, due to errors of the GSEA FDR estimations introduced by the overall inflated candidate PAG count of PAGER 2.0 for GSEA evaluations (PAGER 2.0: 18 136 candidate PAGs vs PAGER-CoV: 4 612 candidate PAGs).

In the original study of GSE147507, the authors reported a unique transcriptional response of cells infected with SARS-CoV-2 unique from other known respiratory viruses, namely, a markedly subdued interferon-I and -III expression as well as higher chemokine expression (most notably IL-6). Our GSEA PAGER-CoV-GSEA case study results are consistent with these findings because we observed significant enrichment of the PAGs relating to 1) cytokine response and inflammation (WIG000864, WIG001072 and WIG000005), in Set B2, 2) NF-kB signaling (WIG000733 in Set B1; FEX000120 in Set C), and 3) other immune pathways upstream of IL-6 expression (WIG001050 in Set B2; WAG000055 in Set C; and FAX000905 in set B1). Interestingly, three PAGs of high significance relating to the nervous system (WIG000823, FEX000140, WIG000048) from three unique data sources (WikiPathways, GeneSigDB, Reactome) were enriched in the PAGER-CoV-GSEA, suggesting strong biomolecular mechanistic links between COVID-19 and damage to the nervous system as reported by (43).

## DISCUSSION

In this work, we describe the development of a comprehensive coronavirus-related gene set database for functional genomic downstream studies. With the continued influx of genomic and functional data, PAGER-CoV database content will need to be periodically updated. We expect the update will primarily be based on the framework described earlier to include both manual curated PAGs from literature and automatically imported PAGs from gene set databases with refined search terms. To make the database truly useful, future developers must consider the delicate balance between comprehensive coverage, the data quality, and potential impact on GSEA analysis recall performance among candidate PAGs. While we designed the database web user interface to be minimalistic for ease of navigation, we plan to introduce additional database features, e.g., reference data source links, additional PAG curation, and links to applications for network visual analytics, as this resource grows it's user base.

## DATA AVAILABILITY

PAGER-CoV is freely available to the public without registration or login requirements (http://discovery.informatics.uab.edu/PAGER-CoV/). The data is available for download based on the agreement of citing this work while using the data from PAGER-CoV website.

## Supplementary Material

gkaa1094_Supplemental_Files
